# Mental health monitoring in the workplace using artificial intelligence: A scoping review

**DOI:** 10.1371/journal.pdig.0001743

**Published:** 2026-09-21

**Authors:** Kalee Jack, Julie Tian, Viktoriia Kurkova, Setayesh Modanloo, Liz Dennett, Jasmine Noble, Andrew Greenshaw, Jake Hayward

**Affiliations:** 1 Department of Psychiatry, Faculty of Medicine & Dentistry, University of Alberta, Edmonton, Alberta, Canada; 2 Geoffrey and Robyn Sperber Health Sciences Library, University of Alberta, Edmonton, Alberta, Canada; 3 Department of Emergency Medicine, Faculty of Medicine & Dentistry, University of Alberta, Edmonton, Alberta, Canada; The Second Affiliated Hospital of Chongqing Medical University, CHINA

## Abstract

Workplace mental health issues are a prevalent concern. A potential solution is remote health monitoring. In this scoping review, we map evidence on artificial intelligence (AI) for workplace mental health monitoring. MEDLINE, PsycINFO, Scopus, Embase, and Web of Science were searched until March 2024. Studies that evaluated AI for monitoring of a common workplace mental health concern in an actual workplace setting were included. Thirteen studies were included; most (8/13, 62%) were conducted in an office environment, and 4/13 (31%) were in healthcare. Most studies monitored stress (10/13, 77%). The most common method was wearable sensors (8/13, 62%). Studies monitored a wide variety of parameters; the most common were physical activity (7/13, 54%), heart rate or heart rate variability (7/13, 54%). A wide variety of participant-report surveys, including validated (7/13, 54%) and non-validated (6/13, 46%) surveys, were used to measure mental health outcomes. Studies employed diverse AI methods, including supervised, semi-supervised and other (Markov chain and Monte Carlo). The most common AI models used were support vector machines (5/13, 38%), decision trees (5/13, 38%), naive Bayes (4/13, 31%), and random forest (4/13, 38%). Model accuracy ranged from 62%-99%. Future work should identify standard metrics to enable cross-comparability and use larger, high-quality datasets for enriched training, advanced optimization techniques, and careful model tuning.

## Author summary

Workplace mental health is an important concern for both employees and employers. Remote health monitoring with artificial intelligence (AI) can help to detect issues and prompt intervention. This review mapped existing evidence sourced from a systematic search of online databases until March 2024. We included studies that evaluated AI for monitoring of a common workplace mental health concern in an actual workplace setting. Thirteen studies were included. The most common workplaces were an office (8/13, 62%) and healthcare (4/13, 31%). Stress was the most frequently monitored mental health concern (10/13, 77%). The most common method was wearable sensors (8/13, 62%). A wide variety of AI methods were applied. Model accuracy ranged from 62%-99%. Overall, we found heterogeneity across studies and methods, which limits generalizability. Our findings suggest potential for AI-based workplace mental health monitoring which could improve workplace mental health. Standardization of methods and definitions is a priority for future work, as well comprehensively describing practical challenges and methodological limitations associated with the implementation of this technology in the workplace setting.

## Introduction

In the workplace, mental health conditions are a leading cause of disability [1]. Poor workplace health and wellness increases the risk of depression, anxiety, stress, burnout, substance use disorders, among other illnesses, and contributes to increasing financial and human costs, including compromised physical health and quality of life, and increased risk of stigma and discrimination [2–10]. In Canada, the magnitude of the issue is immense. Approximately one in five individuals [11] are believed to be currently experiencing a mental illness, and the estimated related productivity imposes societal costs about $6.3 billion each year [12]. In reflection of the weight and severity of this issue, the WHO continues to promote workplace mental health supports as a global priority [13,14].

New digital health technologies are creating novel opportunities to monitor and improve health in non-hospital settings, including work environments. For example, remote health tracking using wearable sensors on smartwatches and smartphones can detect real-time changes in physiological parameters that correlate with health outcomes, such as activity, sleep, heart rate, body temperature, electrodermal activity, respiration rate, and more [15–17]. These sensors generate enormous amounts of data at the which, if processed efficiently, could inform proactive, ‘just-in-time’ health interventions for employees. When combined with other types of longitudinal data like surveys (including ecological momentary assessments) [18], environmental monitors (e.g., humidity, noise level, and temperature sensors) [19], and voice analysis, a holistic digital representation of individual health emerges [20] that may enable preventative care to improve health outcomes and reduce healthcare utilization [21,22]. However, processing these novel data types with traditional techniques is challenging [23]. Recently, advancements in artificial intelligence (AI), including machine learning, have made meaningful analysis of personal device data more feasible. Combing sensor data with AI techniques has shown promise in remote monitoring of diabetes, hypertension, atrial fibrillation, depression, anxiety, psychosis, stress and more [24].

While prior commentaries and reviews have discussed the general application of AI in the mental health realm [25,26], none have specifically focused on the workplace setting. This scoping review aims to systematically describe current literature on use of AI for personalized mental health monitoring in the workplace to identify knowledge gaps and provide a comprehensive overview of an emerging field.

## Methods

We followed Preferred Reporting Items for Systematic reviews and Meta-Analysis Protocols for Scoping Reviews (PRISMA-ScR) guidelines and the PRISMA for Searching Extension for this scoping review [27]. A protocol was prospectively registered in Open Science Framework (https://doi.org/10.17605/OSF.IO/PF4MB).

### Eligibility criteria

We followed the PCC (Population, Concept, and Context) framework for scoping reviews, as recommended by the Joanna Briggs Institute [28] when developing our eligibility criteria, which are summarized in Table 1. Included studies evaluated the use of AI for monitoring mental health (e.g., deep learning, machine learning, neural network), with participants 18 years of age and older. We focused on the adult population, as most of the workforce is adults. As they are the most prevalent and relevant mental health issues in the workplace, studies were limited to those involving depression, anxiety, stress, burnout, post-traumatic stress disorder (PTSD), or substance use disorder [2–7].

**Table 1:** Eligibility criteria for this review, which were based on the PCC (Population, Concept, and Context) framework for scoping reviews, as recommended by the Joanna Briggs Institute for Scoping reviews [28].

	Inclusion criteria	Exclusion criteria
Population	• Adults (18 years of age and older)	• Participants under the age of 18
Concept	• The use of AI (e.g., deep learning, machine learning, neural network) to monitor a mental health concern of a worker (depression, anxiety, stress, burnout, PTSD, substance use disorder) • Monitoring: more than 1 measurement over a period of time	• A mental health concern that is not listed in the inclusion criteria (schizophrenia, personality disorders) • Physical health concerns • Studies focused on health care professionals addressing patient concerns with AI
Context	• Must be in a real-world workplace setting • All participants must be employees and the study must clearly be in a workplace setting	• School or educational setting • Experimental laboratory setting
Language	• Can be translated to English using google translate	• Cannot be translated to English using google translate
Publication type/study design	• Conference abstracts/presentations • randomized controlled trials, longitudinal studies and observational studies	• Reviews • Preprints • Cross-sectional studies • Proof of concept • Studies using external datasets

The context for this review was set to workplaces, thus, only studies that studied AI monitoring with workers in a real-world, in-person workplace settings were included. Studies conducted in controlled laboratory simulations were excluded. We included studies on trainees (e.g., residents, provisional psychologists, practicum teachers), provided that the study took place in a workplace setting. If studies could not be translated to English using Google Translate, they were excluded. Studies had to include a monitoring in a real-world workplace setting with real workers, so we excluded proof of concept studies that discussed theory but did not actually implement monitoring using AI. Conference proceedings, randomized controlled trials, longitudinal studies and observational studies were included. Reviews, preprints, cross-sectional studies, and studies that used external datasets were excluded. External datasets were excluded because the focus was on studies done in a real-world setting [28].

### Search strategy

We searched 5 electronic databases: MEDLINE (1946-present via OVID), PsycINFO (1967-present), Scopus (2004-present), Embase (1974-present), and Web of Science (1997-present) from inception to March 2024. We identified relevant seed articles for the search and used those seed articles to help identify the search concepts and build the final search strategy. The search includes an extensive list of subject headings and keywords for 4 concepts: wearables, health monitoring (to differentiate from general fitness use), outpatient/remote (to differentiate from in-hospital monitoring) and physician attitudes. Terms for each concept were combined with Boolean OR and the four concepts were combined together with Boolean AND. Terms in the title of articles that implied fitness uses or hospitalized patients were also removed from the search in a final step. S1 Text is a detailed version of our Medline search strategy.

### Study selection

Following our electronic database search, studies were uploaded into Covidence Systematic Review software [29]. Covidence was used to screen studies; two reviewers independently screened studies at both the title/abstract and full-text review stages, and a third reviewer resolved conflicts.

### Data extraction and synthesis of results

Data extraction was completed independently by two reviewers using a spreadsheet that was prepared a priori by the authors. A third reviewer resolved any conflicts. We extracted data on study characteristics (country, publication type, sample size, participant age), workplace setting, mental health issue of interest, monitoring method, parameter monitored, monitoring frequency and length, AI method used, performance evaluation, and AI model limitations. Percents and percent proportions were calculated to summarize the results.

## Results

### Search results and study selection

**Fig 1:**
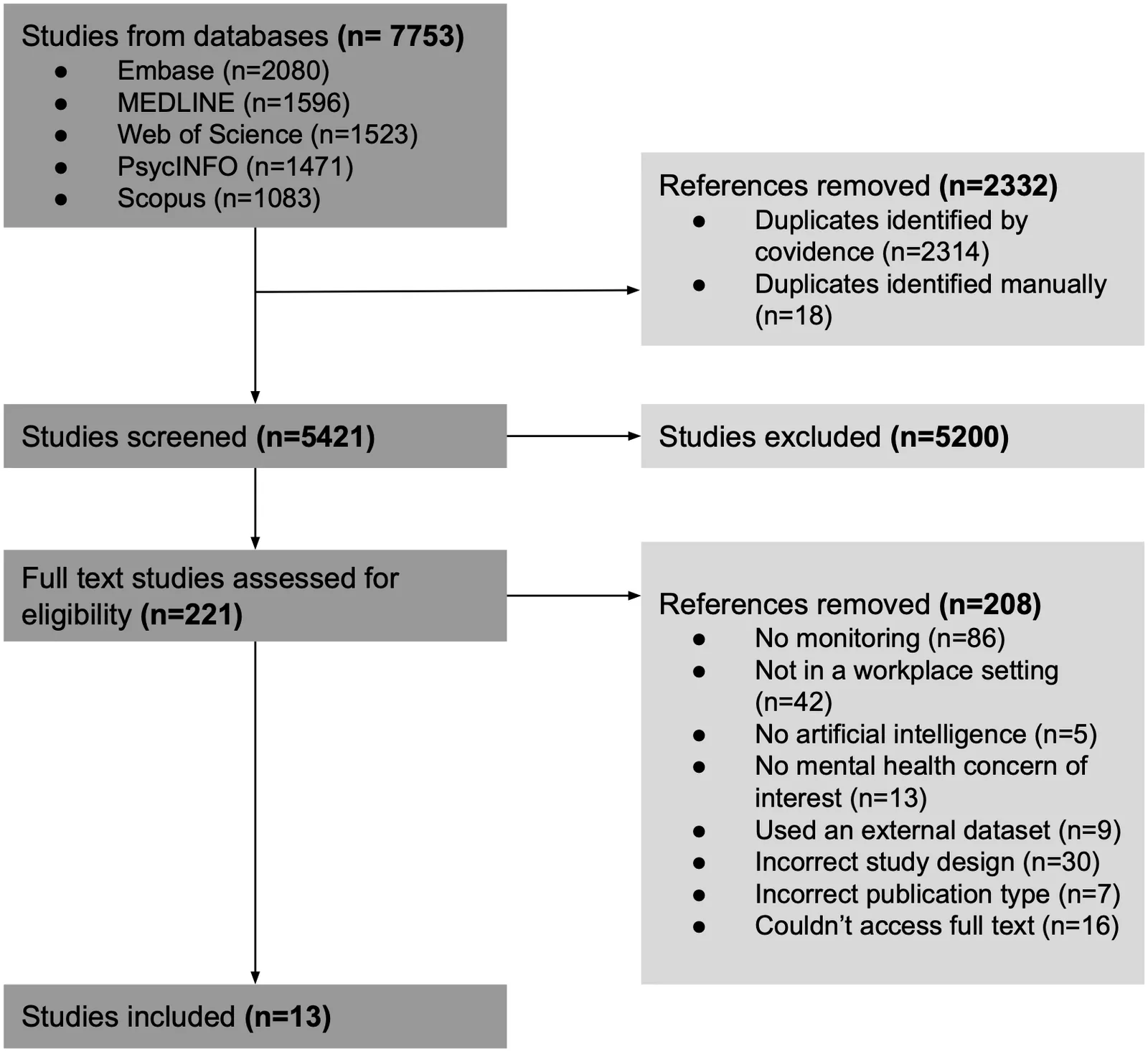
PRISMA flow diagram showing the search results and study selection process.

The results of our search and our study selection process is shown with a PRISMA flow diagram (Fig 1). Altogether, our search identified 7753 studies: 2080 from Embase, 1596 from MEDLINE, 1523 from Web of Science, 1471 from PsycINFO and 1083 from Scopus. After duplicates were removed, 5421 studies remained that were eligible for title and abstract screening. At this stage, 5200 studies were excluded, leaving 221 studies that were assessed for eligibility using the full text. Following full text screening, 13 studies remained [30–42], which was the final number of studies included in the review.

### Study characteristics

Table 2 presents a summary of the 13 studies. Eight of the 13 studies (62%) [30–33,35,38,41,42] were studies published in peer reviewed journals, and the remaining 5 studies (38%) [34,36,37,39,40] were conference proceedings. There were a wide variety of sample sizes, ranging from 8 [35] to 1032 [42].The most common country was the USA (4/13, 31%) [31,34–36] followed by Canada (2/13, 15%) [33,37] and Italy (2/13, 15%) [32,38] Most studies were conducted in an office environment (8/13, 62%) [30–32,34,38,39,41,42]; a few studies took place in a healthcare setting (4/13, 31%), [33,35,36,40]; and 1 study was on police officers (1/13, 8%) [37].

**Table 2:** Summary table of the 13 included studies.

	Publication type / study design	Sample size	Country	Participant age	Occupation	Mental health Outcome / measurement frequency and length	Parameter monitored / method	AI method	Evaluation Metrics of AI Model (For studies with multiple models, the best metric is reported)
**Banholzer et al., 2021** [30]	Journal publication / observational	70	Switzerland	20-61, 39.5*	Tech company employees	Stress / Surveys (7-point Likert) times per day, 7 weeks	Mouse movements, self-reported stress / computer mouse	Bayesian approach, Markov chain, Monte Carlo	There was a negative association between stress and the two-way interaction term of mouse speed and accuracy (mean −0.32, 95% highest posterior density interval −0.58 to −0.08), which means that stress was associated with a speed-accuracy trade-off
**Booth et al., 2022** [31]	Journal publication / observational	606	USA	–	Information workers	Stress / Surveys (5-point Likert, validated) daily for 56 days	Physical activity, phone usage, sleep, heart rate / Garmin Vivosmart 3	Elastic net and random forest algorithms, feed-forward multi-layer perceptron, gated recurrent-unit networks, long short-term memory networks	The random forest yielded the highest F1-score of 0.75 Accuracy (0.62), precision (0.65), recall (0.89)
**Garcia-Ceja et al., 2016** [32]	Journal publication / observational	30	Italy	37.5 + /- 7.3	Private company workers	Stress / Surveys (derived from the OLBI^a^) 3 times per day, 8 weeks	Physical activity, stress / triaxial accelerometer from Samsung Galaxy SIII Mini	Naive Bayes, decision trees, ordinal naive Bayes, random forest model	User-specific model performed best Random classifier ACC1 = 0.86
**Geoffrion et al., 2023** [33]	Journal publication /	816	Canada	–	Healthcare workers	Anxiety, depression, PTSD / Several surveys (GAD-7^b^, PHQ-9^c^, PCL-5^d^) weekly, 12 weeks	Anxiety, depression, PTSD / repeated surveys	Logistic regression, support vector machines	Logistic regression model: over 0.80 accuracy, sensitivity, and specificity 3 week measures: best accuracy and sensitivity
**Hernandez et al., 2011** [34]	Conference proceeding / case study	9	USA	–	Call center employees	Stress / surveys (7-point Likert) after each call, 1 week	Skin conductance / wristband biosensor	Support vector machines	Best model: Precision: 78%
**Kaczor et al., 2020** [35]	Journal publication / observational	8	USA	34-60 (42.9)	Physicians	Stress / written log and survey (descriptions of stressful events and overall stress) 9 clinical shifts typically 8–10 hours in duration, 6 months	3D accelerometry, EDA, skin temperature, heart rate variability / Empatica E4 device	Linear discriminant model, bagged trees model, kernel naive Bayes model	Best model: Sensitivity: 74%, specificity: 63%, accuracy: 69%, AUC: 0.72
**Liu et al., 2022** [36]	Conference proceeding / observational	88	USA	–	Interns and resident physicians	Burnout / 6 surveys (PFI^e^) every 4 weeks	Burnout status based on workload exhaustion and depersonalization / electronic health record activity logs	HiPAL framework, hierarchical predictive model, semi-supervised framework	Best model: accuracy: 0.65 (Semi-HiPAL-r)
**Marois et al., 2018** [37]	Conference proceeding / observational	19	Canada	–	Police officers	Stress / surveys (10-point Likert) every 15 minutes, 54 work shifts	Heart rate, heart rate variability, respiratory rate, activity, core temp, stress level / Zephyr BioModule BH3 chest strap	Multifactorial logistic regression, decision tree model	Best model: Multifactorial logistic regression accuracy of 66%, decision tree model accuracy of 79%
**Maxhuni et al., 2021** [38]	Journal publication / observational	30	Italy	37.5	Private company workers	Stress / surveys (OLBI^a^, POMS^f^) 3 times per day, 8 weeks	Occupational health, mood, stress (physical activity using location, phone conversations, SMS logs, preinstalled system, social apps) / Samsung Galaxy S3 mini 32 GB; 3-axial linear acceleration	Semi-supervised learning that uses a single classifier called Self-Training	Male achieved better accuracy: 72% (Precision: 74%; Recall: 79%) for supervised approach and 76% (Precision: 74%; Recall: 79%) for SSL, in contrast to Female with 60% (Precision: 59%; Recall: 60%) for supervised and 65% (Precision: 62%; Recall: 65%) for SSL approach
**Nishimura et al., 2022** [39]	Conference proceeding / observational	100	Japan	42.1	Office workers	Anxiety, depression, stress / surveys (DAMS^g^) 2 weeks	Calorie consumption, heart rate, sleep characteristics, metabolic equivalent, step count, floor count, activity characteristics, behavioral data of what they were doing and when, weather data / Fitbit	Light Gradient Boosting Machine	The prediction accuracy and f1 scores for all psychological indicators were 72–89%
**Sakib et al., 2023** [40]	Conference proceeding / observational	12	Bangladesh	27-50	Nurses	Stress / surveys (7-point Likert) 1 week	Accelerometer data / 3D accelerometer sensor ADXL345 and an Arduino UNO	Nearest Neighbor Classifiers, Neural Networks, Support Vector Machines, Naive Bayes, Discriminant Analysis, Decision Trees, Ensemble Classifiers	The MG-SVM model achieved promising performance for all classes. Accuracy 80%, Sensitivity 81%, Specificity 80%, Precision 79%, Recall 78%, F-score 80%
**Soto et al., 2021** [41]	Journal publication / observational	14	–	40	Knowledge workers	Stress / surveys (5-point Likert) 3 times per day, 8 weeks	Physical activity (intensity of motion, energy expenditure, step counter), heart rate, blood pulse wave, heart rate variability, blood oxygenation, blood perfusion, galvanic skin response, skin temperature, respiratory rate; stress level through surveys / Biovotion's Everion biometric sensors	Random forest, Naïve Bayes, decision trees, support vector machine, and a multilayer perceptron neural network	Random forest stress prediction accuracy 0.77, precision 0.32, recall 0.26
**Vincent et al., 2021** [42]	Journal publication / observational	1032	–	38	IT professionals	Depression / survey (HAM-D^h^) About 2 weeks	Heart rate recorded average from sensors / smart band	Deep multi-layered perceptron with and without backpropagation, SVM (linear), random forest ensemble (SVM, random forest)	In all the three evaluation metrics used, the deep-MLP model with backpropagation outperforms the other. Accuracy 0.99

*median age.

^a^Oldenburg burnout inventory.

^b^General Anxiety Disorder-7.

^c^Patient Health Questionnaire.

^d^short form of the Post-Traumatic Stress Disorder Checklist for Diagnostic and Statistical Manual of Mental Disorders, fifth edition.

^e^Stanford Professional Fulfillment Index.

^f^Profile of Mood States.

^g^Depression and Anxiety Mood Scale.

^h^Hamilton rating scale for depression.

### Monitoring method and parameters

Table 2 shows the monitoring method and AI model accuracy for each of the 13 included studies. Studies used a wide variety of monitoring methods; the most common method was wearable sensors (8/13, 62%) [31,34,35,37,39–42]. Each study used a different wearable sensor, including the Garmin Vivosmart 3 [31], a wristband biosensor [34], the Empatica E4 device [35], the Zephyr BioModule BioHarness 3 chest strap [37], a Fitbit [39], an ADXL345 and an Arduino UNO [40], Everion biometric sensor [41], and a smart band (brand not mentioned in the paper) [42]. Other monitoring methods included the Samsung Galaxy SIII Mini smartphone (2/13, 15%) [32,38], a computer mouse (1/13, 8%) [30], and electronic health record activity logs (1/13, 8%) [36]. Like the monitoring method, the monitored parameters were also highly variable across studies (Table 2). The most common were physical activity (including accelerometry data and step count) (7/13, 54%) [31,32,35,37,39–41] and heart rate or heart rate variability (7/13, 54%) [31,35–37,39,41,42]. Other parameters were skin or core temperature (4/13, 31%) [35–37,41], respiratory rate (3/13, 23%) [36,37,41], sleep (2/13, 15%) [31], mouse movements (1/13, 8%) [30], calorie consumption (1/13, 8%) [39], behavioral data (1/13, 8%) [39], phone usage (1/13, 8%) [31], sleep [31,39], blood oxygenation [41], blood perfusion [41], galvanic skin response [41], occupational health [38], and electrodermal activity [35].

### Measuring mental health outcomes

The included studies used surveys to measure mental health outcomes. Studies employed a variety of surveys at many different frequencies. Some studies did not use validated scales in their surveys (6/13, 46%) [30,34–36,40,41]; Likert scale questions were used, as well as descriptions of stressful events (6/13, 46%) [30,34,35,37,40,41]. The remaining studies used validated scales in their questionnaires (7/13, 54%), including a validated 5-point Likert scale [31], the OLBI (Oldenburg burnout inventory) [32,38] the GAD-7 (General Anxiety Disorder-7) [33], the PHQ-9 (Patient Health Questionnaire) [33], the PCL-5 (short form of the Post-Traumatic Stress Disorder Checklist for Diagnostic and Statistical Manual of Mental Disorders, fifth edition) [33], the PFI (Stanford Professional Fulfillment Index) [36], the POMS (Profile of Mood States) [38], the DAMS (Depression and Anxiety Mood Scale) [39], and the HAM-D (Hamilton rating scale for depression) [42]. Most of the 8 studies with repeated surveys distributed these surveys more than once per day (5/8, 63%) [30,32,37,38,41]. One study had surveys once per day (1/8, 13%) [31], and 2 studies had a survey frequency of less than once per day (2/8, 25%) [33,36]. The monitoring length of the included studies ranged from 1 week [40] to 1 year [31]. The most common monitoring timeframe was less than 3 months (9/13, 69%) [30,32–34,38–42], followed by 3 months to 6 months (2/13, 15%) [35,36]; one study monitored for over 6 months (1/13, 8%) [31].

### AI models and mental health concerns

**Fig 2:**
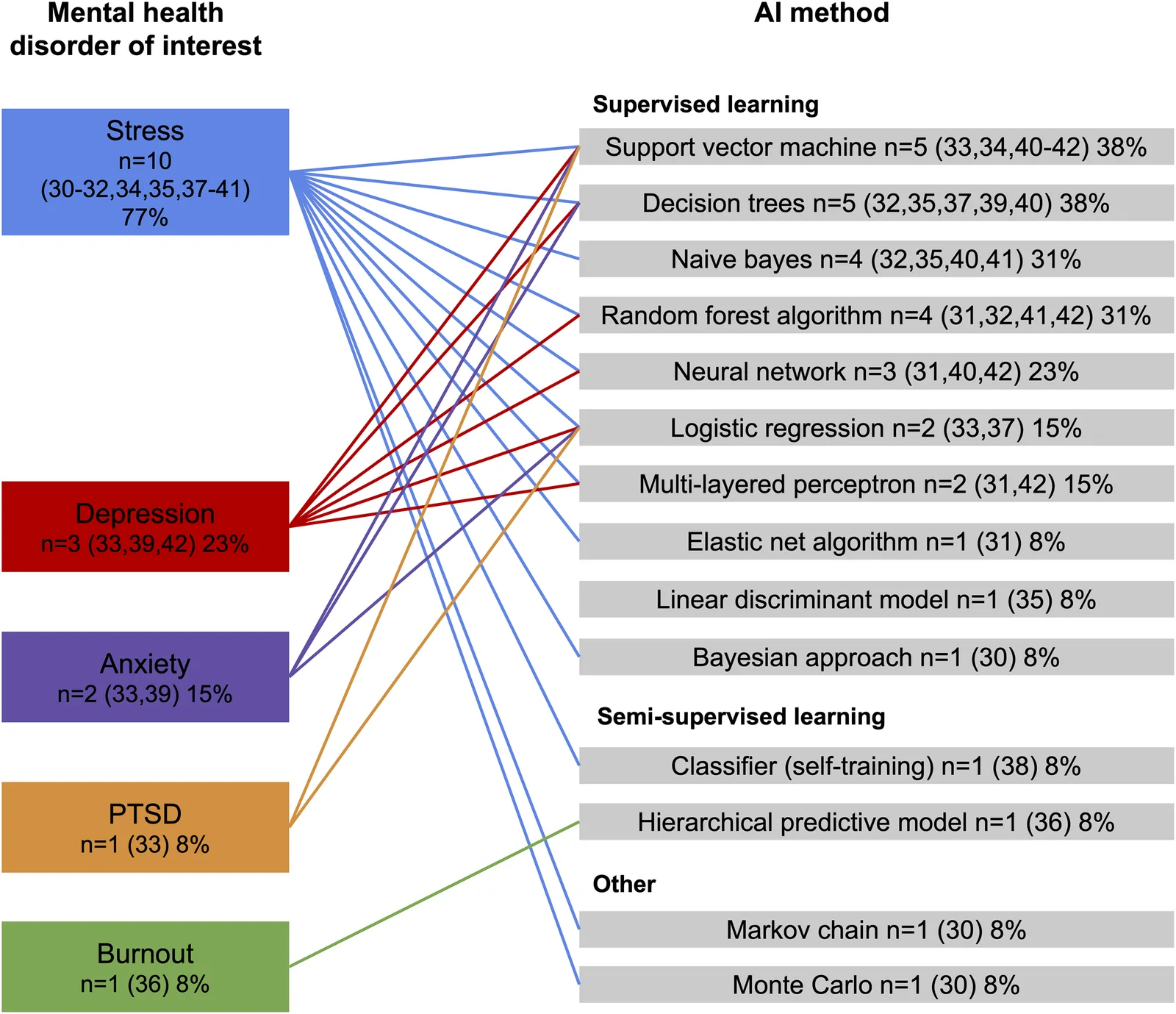
AI methods used in the included studies categorized by mental health disorder of interest.

The most common mental health concern of the included studies was stress (10/13, 77%) [30–32,34,35,37–40,42]. Studies also focused on depression (3/13, 23%) [33,39,42], anxiety (2/13, 15%) [33,39], PTSD (1/13, 8%) [33] and burnout (1/13, 8%) [36] (Fig 2). Studies used many different AI methods (Fig 2), including supervised learning, semi-supervised learning and other AI methods. The most frequently used supervised learning methods were support vector machine (5/13, 38%) [33,34,40–42] and decision trees (5/13, 38%) [32,35,37,39,40], followed by naive Bayes (4/13, 31%) [32,35,40,41], random forest algorithm (4/13, 31%) [31,32,41,42], neural network (3/13, 23%) [31,40,42], logistic regression (2/13, 15%) [33,37] multi-layered perceptron (2/13, 15%) [31,42], elastic net algorithm (1/13, 8%) [31], linear discriminant model (1/13, 8%) [35] and Bayesian approach (1/13, 8%) [30]. Semi-supervised AI methods included single classifier (1/13, 8%) [38] and a novel hierarchical predictive model (1/13, 8%) [36]. Other AI methods were Markov chain (1/13, 8%) [30] and Monte Carlo (1/13, 8%) [30]. Fig 2 shows the types of AI that were used for different mental health issues of interest. All included studies described the model metrics used to evaluate the ML models used for the studies. Examples of metrics used to evaluate model performance include accuracy, F1, AUC curves, sensitivity, and specificity. Table 2 shows the highest reported AI model accuracy for each study. Accuracy ranged from 62% [31] to 99% [42]. Of the 11 studies that reported AI model accuracy (11/13, 85%) [31,33–42], most studies had an accuracy greater than 75% (8/11, 73%) [32,33,37–42]. Around one third of the studies (4/13, 31%) [33,35,36,41] included statements about AI model limitations. Limitations mainly revolved around data quality, data quantity, and modeling optimization.

## Discussion

Our review explores applications of AI for mental health monitoring in the workplace, identifying 13 studies for comprehensive analysis after a rigorous screening process. The geographical distribution of these studies showed a predominance from the USA (31%), followed by Canada and Italy (15% each). Most studies were conducted in office environments (62%), with a notable proportion in healthcare settings (31%), reflecting the diverse workplace contexts in which mental health monitoring is applicable. Sample sizes varied significantly, ranging from 8 to 1032 participants. The studies employed various monitoring methods, with wearable sensors being the most common (utilized in 62% of studies). A wide range of wearable sensors were tested, highlighting large heterogeneity in technical implementations. Commonly monitored parameters included physical activity (54%) and heart rate variability (54%). Monitoring durations ranged from one week to one year, with most studies monitoring for less than three months, suggesting a preference for short-term study designs. In the realm of mental health, stress (77%) was the most common outcome studies, with other studies addressing depression (23%), anxiety (15%), PTSD (8%), and burnout (8%). This emphasis aligns with the high prevalence and impact of stress-related disorders in modern workplaces and healthcare environments [7,35].

Our scoping review aligns with existing literature, revealing that stress was the most frequently monitored mental health issue [43]. This prevalence underscores the significant impact of workplace stress and the urgent need for effective monitoring and intervention strategies. Alternatively, studies may have focused on stress since this is less stigmatized than other mental health disorders. Job strain, stress, and long working hours, significantly increases the risk of cardiovascular issues like coronary heart disease and stroke [44]. The recent COVID-19 pandemic further highlighted how exposure to potentially traumatic events paired with work-related stress can trigger episodes of mental health problems such as anxiety, depression, and PTSD [45]. Moreover, occupational stressors translate into decreased organizational efficiency, higher staff turnover, increased sick leave, a decline in overall work quality and quantity, rising healthcare costs, and ultimately, lower employee satisfaction [46].

The variability in monitored parameters across studies, such as physical activity, heart rate variability, self-reported stress, and sleep, underscores the multifaceted nature of mental health and the importance of a holistic monitoring approach. Traditional methods for diagnosing psychiatric disorders rely on in-person assessments in clinic. A reliance on episodic assessment can be problematic because mental health characteristically varies over time and patients can have trouble accurately recalling past events and symptoms [47]. Modern wearable devices, which were a common data source in our review, offer potential for tracking changes in mental health metrics over time and providing real-time feedback to help patients and clinicians identify behavioral patterns, symptom triggers and potential treatment strategies [48]. However, we found large heterogeneity across studies in terms of sensors, biometrics, and outcomes making cross-study comparisons and synthesis of results challenging. Future work should focus on standardizing metrics and outcome definitions to support comparison of results and synthesis of data.

While wearables provide valuable data, they do not capture the full picture of mental health. Wearables miss nuanced emotional or psychological states, such as feelings of anxiety, stress, or specific cognitive symptoms like racing thoughts or difficulty concentrating, which are better captured through self-reports or clinical assessments [49]. Our results underscore this gap, as most included studies (8/13) employed repeated surveys to capture subjective mental health measures, with 63% of these studies administering surveys more than once per day. These surveys were critical for obtaining data on emotional and cognitive symptoms that wearable devices alone could not detect. Additionally, the use of AI models in the included studies proves that combining data sources enhances predictive capabilities. For instance, supervised learning methods like support vector machines and decision trees were frequently employed to process both wearable and survey data, achieving high accuracy levels (75% or higher in 7/11 studies reporting accuracy).

The included studies employed a variety of AI methods to address mental health issues. Supervised learning techniques were most common, with support vector machines and decision trees each employed in 38% of the studies. These methods are known for their robust classification task abilities and have been widely used in health research [50,51]. Other notable supervised methods included naive Bayes (31%), random forest (31%), neural networks (23%), and logistic regression (15%), reflecting the various algorithmic approaches available for mental health classification. Semi-supervised and other AI approaches, such as single classifiers and Markov chains, were also explored, though less frequently. The performance metrics for these models varied, with reported accuracies ranging from 62% to 99%. This wide range of accuracy may be attributed to the chosen modeling techniques as well as a variety of study designs, participant demographics, and outcomes of interest [52,53]. Most studies reported accuracy rates exceeding 75%, indicating the potential effectiveness of these models in real-world applications. However, the limitations noted across the studies, including issues with data quality, data quantity, and the need for better modeling optimization, emphasize the critical need for high-quality, large datasets and advanced optimization techniques to improve the reliability and generalizability of machine learning models in mental health research [54]. These high performance values may also be misleading when considered without validation approaches, sample sizes, and risks of overfitting. Given these limitations, the practical significance of these models may be restricted.

Developing machine learning models for health status prediction presents several challenges to researchers, yet only four studies in our review explicitly discussed these limitations. Soto highlighted the risk of overfitting in a small dataset [41]; Kaczor et al. described that poor data quality might obscure stress measurement [35]; Liu et al. noted difficulty in obtaining an adequately labelled dataset for burnout prediction [36]; Overall, we find that the challenges and limitations of AI applications in health monitoring were poorly addressed in extant studies. In general, large, accurate, complete, labelled datasets are known to be crucial for training robust models in AI and obtaining these data can be a massive challenge and barrier to research and innovation [55]. Further, supervised learning techniques often depend on features manually selected by researchers [56], which can introduce bias that negatively impacts model performance. Future work in this domain should describe the practical and technical barriers to AI implementation in real world settings, including data collection methods, ethical concerns related to data bias, and optimizing model performance through techniques like hyperparameter tuning and feature engineering [23].

Although this review focuses on mapping the evidence for the application of mental health remote monitoring using AI in workplace settings, there are important ethical implications that must be considered from a policy, research, and implementation perspective before this technology is widely adopted in real workplace settings. This technology could widen the power gap between employees and employers, which impairs the ability of employees to provide true informed consent to collection of their health data [57,58]. There is potential for issues around privacy, data ownership, and surveillance [58]. Furthermore, mental health remote monitoring could exacerbate discrimination of employees who live with mental illness [58,59]; on the other hand, it could create opportunity for early mental health concern identification and intervention [60]. It will be important to have established regulatory frameworks to protect employee rights prior to the implementation of this technology [58].

## Limitations

Our scoping review has several limitations. Scoping reviews focus on summarizing a broad variety of literature, thus compromising depth of the results [61]. There is high variability in terminology used in this field [62], so it was difficult to generate a comprehensive search strategy. We found a balance between comprehensiveness and practicality, so we could deliver a timely review in this rapidly evolving field. We searched databases that included grey literature, but it’s possible that some was missed. In alignment with scoping review guidelines [61], we focused on the breadth of evidence rather than the quality, but some included studies had relatively small sample sizes and limited monitoring periods, which may impact the generalizability of our results. We focused on mental health conditions with the highest prevalence and relevance to the workplace, which may limit generalizability to excluded conditions such as schizophrenia. Most studies focused on stress, which may also limit generalizability to all mental health conditions.

## Conclusions

Workplace mental health may be improved through use of personalized data combined with AI techniques for individualized health and wellness monitoring. Our review identified a wide array of studies supporting the use of AI methods combined with personalized data, including surveys and wearable sensors. The most frequently measured mental health issue was stress. While findings seem to suggest overall potential for AI-based personal health monitoring in the workplace, wide heterogeneity across studies and a lack of methodological standardization reduces generalizability and applicability of results. Future work should seek to refine and standardize methods/definitions and include a more fulsome description of the practical challenges and methodological limitations associated with ML/AI implementation in the workplace setting.

## Supporting information

S1 Text MEDLINE search strategy. (DOCX)

S1 Checklist PRISMA checklist. (DOCX)
