# Supplementary material for: Mental health monitoring in the workplace using artificial intelligence: A scoping review
Source: PLOS Digit Health. 2026 Sep 21;5(9):e0001743. doi: 10.1371/journal.pdig.0001743 (PMC13592608; doi:10.1371/journal.pdig.0001743)
Supplement: Supplementary file 1 [file pdig.0001743.s001.docx]

**S1 Text.** MEDLINE search strategy

Ovid MEDLINE(R) ALL <1946 to March 20, 2024>

Date searched: March 21, 2024

1	Mental Health/	66245

2	mental disorders/ or exp anxiety disorders/ or mood disorders/ or exp depressive disorder/ or substance-related disorders/ or exp alcohol-related disorders/ or amphetamine-related disorders/ or cocaine-related disorders/ or marijuana abuse/ or exp narcotic-related disorders/ or substance abuse, intravenous/	636092

3	occupational stress/ or burnout, professional/	20996

4	Stress, Psychological/	134899

5	("mental health" or "mental wellness" or "mental wellbeing" or "mental well-being" or "mental* ill*" or "mental disorder*" or "psych* health" or "psych* illness*" or "psych* disorder*" or "psych* assessment*" or "psych* evaluation*" or "psych* wellness" or "psych* well-being" or "psych* wellbeing" or "mental resilience" or "panic attack*" or (resilience adj6 (stress or health)) or depression or depressive or anxiety or stress-level* or "high* stress*" or "psych* stress*" or "psych* distress" or "mental stress*" or "mental distress*" or "occupational stress*" or "job stress*" or "work stress*" or burnout or "stress disorder*" or ptsd or ptsi or post-trauma* or posttraum*).mp.	1346855

6	(((substance* or opioid* or heroin or illicit-drug* or "illegal drug*" or "intravenous drug*" or fentanyl or narcotic* or opiate* or methadone or buprenorphine or benzodiazepine* or benzos or stimulant* or cocaine or methamphetamine* or amphetamine or marijuana or cannabis or hashish) adj3 ("use" or user* or abuse* or usage or misuse* or addict* or disorder* or dependen* or withdrawal)) or "drug abuse").mp.	292472

7	(alcoholi* or ((alcohol or ethanol) adj4 (abuse or addict* or "use" or consum* or drink* or disorder* or misuse or dependen* or withdrawal))).mp.	326784

8	1 or 2 or 3 or 4 or 5 or 6 or 7	1900434

9	workers' compensation/ or Occupational Health/ or Occupational Health Services/ or occupational stress/ or burnout, professional/	73461

10	employment/ or Work/ or work performance/ or Workplace/ or Industry/	131583

11	((worker* not ("healthcare worker*" or "care worker*" or "health worker*" or "social worker*")) or (employee* not ("healthcare employee*" or "health care employee*")) or miners or "mining industry" or "administrative assistant*" or receptionist* or secretaries or "manual labourer*" or "manual laborer*" or "physical labourer*" or "physical laborer*" or ((warehous* or construction or farm or agricultur* or "fast food" or food-service or grocery or store or retail) adj3 (work* or employ* or job* or labor* or labour* or industry)) or steelworker* or ironworker* or "vehicle operator*" or "equipment operator*" or "machinery operator*" or "transit operator*" or "bus driver*" or "taxi driver*" or "truck driver*" or "government employees" or hairdresser* or barber* or barista* or janitor* or cashier* or electrician* or carpenter* or plumber* or welder* or "auto* mechanic*" or "heavy duty mechanic*" or stewardess* or "air steward*" or bartender* or waitstaff or waiter* or waitress or waitresses or bookkeeper* or "air traffic controllers" or bankers or stockbroker* or actuaries or "insurance adjusters" or "insurance brokers" or "claims adjusters" or dental hygienist* or "helping profession*" or "caring profession*" or librarian* or "information specialist*" or journalist or journalists or accountant* or "park ranger*" or (pilots and (airline* or jet or jets or ferry or ferries or plane or planes or airplane* or air)) or "managerial staff" or "sales staff" or salesperson* or "office staff" or firefighter* or firem* or ("public safety" adj3 (professional* or official* or personnel* or staff)) or ("search and rescue" adj3 (team* or member* or crew*)) or ((corrections or correctional) adj (worker* or staff or personnel or official* or officer*)) or "security guard*" or "security personnel" or (border adj3 (agent* or personnel))).mp.	278500

12	((work* not (workflow* or work-flow* or working-memory or "working group*" or workgroup* or workshop* or "social work")) or (occupation* not "occupational therapy") or employ* or job or jobs or staff).ti,bt,kf.	418421

13	(work-related or job-related or employment-related or work environment* or workplace* or work place* or worksite* or work site* or jobsite or job site or "at work" or "on the job" or "while working" or work* compensation or "job stress" or "work stress" or "occupational stress*" or ((professional* or work or job or employment) adj5 burnout)).mp.	154624

14	administrative personnel/ or astronauts/ or correctional facilities personnel/ or counselors/ or farmers/ or government employees/ or laboratory personnel/ or lawyers/ or librarians/ or metal workers/ or military personnel/ or miners/ or exp educational personnel/ or pilots/ or exp religious personnel/ or research personnel/ or social workers/ or truck drivers/	136503

15	("Academic faculty" or "tenure-track faculty" or "faculty members" or professor or professors or teacher* or "education* assistants" or "instructors" or researchers or architect or architects or engineers or attorney* or lawyer or lawyers or paralegal* or "legal assistant*" or "legal professionals" or entrepreneur or entrepreneurs or "web developer*" or "software developer*" or "software engineer" or "software engineers" or "computer programmer*" or "software programmer*" or "systems analysts" or "network administrators" or technicians or scientist* or "research staff" or farmers).ti,bt,kf.	66217

16	exp Emergency Responders/	15968

17	(police* or officer or officers or "peace officer*" or sherriff* or EMT or EMTs or EMS or ("Emergency Medical" adj2 (personnel or technician* or staff)) or paramedic* or "first responder*" or "law enforcement" or "ambulance worker*" or "ambulance staff" or "ambulance personnel" or "emergency manager*" or (("911" or emergency) adj3 (dispatcher or personnel))).ti,bt,kf.	33964

18	exp *health personnel/	449952

19	(psychologist* or psychiatrist* or counselor* or therapist* or physician* or clinician* or doctor* or "general practitioner*" or GP or internist* or specialist* or anaesthetist* or anesthesiologist* or cardiologist* or dermatologist* or diabetologist* or endocrinologist* or gastroenterologist* or geriatrician* or gerontologist* or gynaecologist* or gynecologist* or haematologist* or hematologist* or hepatologist* or immunologist* or neonatologist* or nephrologist* or neurologist* or neurosurgeon* or obstetrician* or ophthalmologist* or oncologist* or orthopedist* or otolaryngologist* or paediatrician* or pediatrician* or pathologist* or podiatrist* or physiatrist* or psychiatrist* or "primary care provider*" or "primary care practitioner*" or pulmonologist* or radiologist* or rheumatologist* or urologist* or nurse or nurses or surgeon* or "social worker*" or dentist* or dietician* or nutritionist* or veterinarian* or "veterinary assistant*" or "veterinary staff" or pharmacist* or audiologist* or "speech language pathologist*" or physiotherapist* or ((health or "health care" or healthcare or care or medical) adj (professional* or worker* or aide* or employee* or personnel or staff)) or "healthcare providers" or "health care providers").ti,bt,kf.	625393

20	exp *Education, Medical, Graduate/ or ((resident* not (permanent resident* or nursing home resident*)).ti,bt,kf. and (residen* education or residen* training or fellowship training or (residen* adj3 (intern or interns or internship*)) or PGE or PGME or ((resident or residents or residency or residencies or intern or interns or internship* or fellow* or graduate or postgraduate or post-graduate) adj4 (medical or medicine or surgical or surgery or anesthesiolog* or anaesthesiolog* or cardiology or dermatolog* or endocrinology or gastroenterolog* or "general practice" or gerontolog* or gynaecolog* or gynecolog* or haematolog* or hematolog* or hepatolog* or immunolog* or neonatolog* or nephrolog* or neurolog* or neurosurg* or obstetric* or ophthalmolog* or oncolog* or orthopedic* or orthopaedic* or otolaryngolog* or otorhinolaryngolog* or paediatric* or pediatric* or pathology or podiatry or physiatry or psychiatr* or pulmonolog* or radiolog* or rheumatolog* or urolog*))).mp.)	70444

21	((psychologist* or psychiatrist* or counselor* or therapist* or physician* or clinician* or doctor* or general practitioner* or GP or internist* or specialist* or anaesthetist* or anesthesiologist* or cardiologist* or dermatologist* or diabetologist* or endocrinologist* or gastroenterologist* or geriatrician* or gerontologist* or gynaecologist* or gynecologist* or haematologist* or hematologist* or hepatologist* or immunologist* or neonatologist* or nephrologist* or neurologist* or neurosurgeon* or obstetrician* or ophthalmologist* or oncologist* or orthopedist* or otolaryngologist* or paediatrician* or pediatrician* or pathologist* or podiatrist* or physiatrist* or psychiatrist* or "primary care provider*" or "primary care practitioner*" or pulmonologist* or radiologist* or rheumatologist* or urologist* or nurse or nurses or surgeon* or "social worker*" or dentist* or dietician* or nutritionist* or veterinarian* or "veterinary assistant*" or "veterinary staff" or pharmacist* or audiologist* or "speech language pathologist*" or physiotherapist* or ((resident or residents) not ("permanent resident*" or "nursing home resident*"))) and ((secondary or vicarious) adj3 (trauma* or stress))).mp.	1546

22	military personnel/	45413

23	("Military personnel" or paramilitary or armed-force* or armed-service* or servicewomen or servicemen or "service member*" or air-personnel or defense-force* or defence-force* or "service personnel" or "army personnel" or "navy personnel" or air-force or marines or sailor* or soldier* or infantryman or Civil-defense or Troops or ranger* or "medic" or "coast guard" or submariner* or "active duty personnel" or "enlisted personnel" or "reserve personnel" or reservist* or "uniformed personnel").mp.	71405

24	or/9-23	1652439

25	artificial intelligence/ or machine learning/ or deep learning/ or supervised machine learning/ or support vector machine/ or unsupervised machine learning/ or latent class analysis/ or random forest/	108558

26	(Artificial intelligence or ((AI or "pattern recognition") adj8 (decision* or monitor* or insight* or algorithm* or tool or tools or analy* or program* or tech* or software or model*)) or computer-assisted or "deep learning" or "machine learning" or "predictive analytics" or "neural network*" or "classification algorithm" or "support vector machine" or "relevance vector machine" or "random forest" or "causal forest" or classifier* or "supervised learning" or "unsupervised learning" or "reinforcement learning" or "computer heuristic*" or "decision tree" or "feature detection" or (Markov adj3 model*) or "learning algorithm*" or ((multifactor* or multicriteria or multi-factor or multi-criteria) adj3 "decision analysis") or "nearest neighbo*" or "neural net*" or "outlier detection" or "network learning" or "transfer learning" or "ensemble learning" or "Bayesian learning" or "gradient boost*" or "clustering algorithm*" or chatbot* or "large language model*" or chatgpt or "generative ai" or ("computer vision" not "computer vision syndrome") or "sentiment analysis" or "probabilistic graphical model*" or "structured probabilistic model*" or "generative adversarial network*" or "natural language processing").mp.	712708

27	25 or 26	715611

28	8 and 24 and 27	1596
